# Pacing-induced cardiomyopathy in chronic right ventricular apical pacing: a midterm follow-up study

**DOI:** 10.1186/s40001-019-0386-5

**Published:** 2019-07-22

**Authors:** Erdal Safak, Hüseyin Ince, Lito Gkouvatsou, Heinz-Peter Schultheiss, Jasmin Ortak, Evren Caglayan, Alper Oener, Giuseppe D’Ancona

**Affiliations:** 1 Vivantes Klinikum im Friedrichshain und Am Urban, Berlin, Germany; 2 Vivantes Klinikum Neukölln, Berlin, Germany; 3 Universitätsmedizin Charité Berlin, Berlin, Germany; 4 Rostock University Medical Center, Rostock, Germany; 5 Department of Cardiology, Vivantes - Netzwerk für Gesundheit GmbH, Klinikum Am Urban, Dieffenbachstraße 1, 10967 Berlin, Germany

**Keywords:** Right ventricular pacing, Cardiomyopathy, Left ventricular ejection fraction

## Abstract

**Background:**

Data concerning the effect of chronic right ventricular pacing in patients with normal left ventricular ejection fraction (LVEF%) are contradictory. The aim of this study is to evaluate the prevalence of pacing-induced cardiomyopathy (PICM) at midterm follow-up after permanent pacemaker implantation (PPM).

**Methods:**

A series of 170 patients were submitted to PPM within our facility. Inclusion criteria were the absence of structural heart disease and a preserved LVEF% (> 45%) at the time of PPM. A midterm clinical and echocardiographic follow-up was performed, and data were collected and analyzed retrospectively. PICM was defined as follow-up LVEF ≤ 45%, dyskinesia during RV pacing, and the absence of other known causes of cardiomyopathy.

**Results:**

At a median echocardiographic follow-up of 24.5 months (IQR 10.0–43.0 months), the overall mean LVEF% decreased from a preimplantation value of 66.7% (± 8.6%) to 63.2% (± 10.6%) (*p* < 0.0001). PICM occurred in 11 patients (6.5%). Patients developing PICM had a significantly lower preimplantation LVEF% (58.4 ± 8.0% vs. 67.3 ± 8.4%; *p* = 0.005), a trend for higher right ventricular pacing time rate (0.7 ± 0.3 vs. 0.5 ± 0.4; *p* = 0.1), a significantly lower rate of PPM indication for sick sinus syndrome (SSS) (18.2% vs. 61.0%; *p* = 0.009), and significantly higher rate of second-grade cardiac conduction block (36.4% vs. 11.3%; *p* = 0.03). At multivariate logistic regression, only preimplantation LVEF% (OR = 0.88; CI 0.80–0.96; *p* = 0.006) and the presence of SSS (OR = 0.1; CI 0.03–0.9; *p* = 0.04) were independently related (inverse relationship) to follow-up PICM.

**Conclusions:**

In this selected PPM patient cohort with preserved LVEF%, the rate of PICM at midterm follow-up is relatively low, but its occurrence seems to be related to baseline LVEF% and PPM indication category.

## Background

There are numerous data showing the adverse hemodynamic effects of a left bundle branch block (LBBB) activation pattern [1–3]. Single-site stimulation in the right ventricle (RV), especially in the RV apex (RVA), creates a LBBB activation pattern resulting in left ventricular (LV) asynchrony similar to that observed in patients with native LBBB [4]. In selected patient subgroups, such as those with low left ventricular ejection fraction (LVEF%) and long QRS duration, LV asynchrony induced by RVA pacing may aggravate a preexisting heart failure [5–11].

Few and controversial data exist concerning the long-term effect of chronic RVA pacing on the systolic LV function of patients with preserved LVEF% at the time of permanent pacemaker (PPM) implantation [12–18].

The aim of this study was to determine the effect of chronic RVA pacing in an unselected typical PPM population with preserved LVEF% and to report the prevalence of PPM-induced cardiomyopathy (PICM) focusing upon its possible independent determinants.

## Materials and methods

Demographic and clinical data of patients undergoing PPM implantation within the premises of the Cardiology Department of Universitätsmedizin Charite Berlin were collected in an electronic chart. The present analysis has been performed retrospectively and has included only those patients for whom the absence of structural heart disease and a preserved LVEF% (> 45%) had been clearly documented at the time of PPM implantation. Patients had signed an informed consent to their treatment and to the use of their data for scientific research purposes.

A pre-procedural and staged follow-up echocardiography (transthoracic echocardiography) was performed in all patients. Baseline and follow-up clinical information was also collected, including ambulatory recordings of the PPM function parameters.

LVEF% was assessed using Simpson’s biplane approach at admission and at the various phases of the echocardiographic follow-up.

### Statistical analysis

Data are presented as absolute numbers, percentages, mean ± standard deviation for normally distributed variables and median with 75% percentile inter-quartile range (IQR) for variables with not-normal distribution.

Follow-up PICM was defined as a combination of LVEF ≤ 45%, dyskinesia during RV pacing, and the absence of other known causes of cardiomyopathy [12].

Patients that did and did not experience follow-up PICM were compared. Differences of preoperative clinical variables and peri-procedural management data (including PPM parameters) were analyzed by means of univariate analysis. Student’s *t* test, Wilcoxon signed-rank test, chi-square test, and Fischer-exact test were used whenever appropriate. A *p* value < 0.05 was considered as significant. A multivariable model was then built, and stepwise backward logistic regression was performed to identify independent determinants for PICM. Starting from the univariate comparison between patients experiencing and not experiencing PICM, we included in the logistic regression model only those variables that at univariate analysis had a statistical difference with a *p* < 0.05. The statistical calculations were run using the SPSS 11.0 software (SPSS for Windows, Chicago, SPSS Inc.).

## Results

Out of 721 patients submitted to PPM implantation within our facility during the period 2007–2011, 170 were included in the present study. All 170 patients underwent successful PPM implantation in the RVA without any relevant procedural complication. At a median echocardiographic follow-up of 24.5 months (IQR 10.0–43.0 months), the overall mean LVEF% decreased from a preimplantation value of 66.7% (± 8.6%) to 63.2% (± 10.6%) (*p* < 0.0001).

PICM occurred in 11 patients (6.5%). No patient was lost at follow-up. Tables 1 and 2 summarize the pre-procedural and follow-up-related information in patients developing and not developing PICM. To summarize, patients developing PICM had a significantly lower preimplantation LVEF% (58.4 ± 8.0% vs. 67.3 ± 8.4%; *p* = 0.005), a significantly higher reduction (delta) of the baseline LVEF% values (20.4 ± 7.9% vs. 2.3 ± 9.9%; *p* < 0.0001), a trend for higher right ventricular pacing time rate at last PPM interrogation (0.7 ± 0.3 vs. 0.5 ± 0.4; *p* = 0.1), a significantly lower rate of PPM indication for sick sinus syndrome (SSS) (18.2% vs. 61.0%; *p* = 0.009), and a significantly higher rate of second-grade cardiac conduction block (36.4% vs. 11.3%; *p* = 0.03). No other significant differences were noticed in the pre-procedural and follow-up findings.

**Table 1:** Preimplantation information for the group without pacemaker-induced cardiomyopathy (non-PICM) and the pacemaker-induced cardiomyopathy (PICM)

Preimplantation	Non-PICM	PICM	*p* value
*n*	159	11	
Age (years ± SD)	71.5 (± 9.7)	69 (± 17)	0.5
Male	58.8%	4.1%	0.6
SSS	61%	18.2%	0.009
AVB2	11.3%	36.4%	0.03
AVB3	23.3%	27.3%	0.7
HTN	80.5%	63.6%	0.24
Diabetes	24.5%	18.2%	0.5
CAD	57.2%	72.7%	0.4
Afib	42.8	18.2	0.13
LVEF (%± SD)	67.3 (± 8.4)	58.4 (± 8.0)	0.005

**Table 2:** Follow-up data (FU)

FU	Non-PICM	PICM	*p* value
SR	13.2%	18.2%	0.64
DR	86.8%	81.8%	0.65
LVEF-FU (%± SD)	65 (± 8.5)	38 (± 4.6)	0.01
ΔLVEF	2.3 (± 9.9)	20.4 (± 7.9)	< 0.0001
RV-PACE%	50 (± 40)	70 (± 30)	0.10
ACEI	56.6%	54.5%	0.84
ARB	30.2%	36.4%	0.74
MRA	75%	25%	0.03
BB	76.1%	81.8%	0.5
CCB	27.7%	9.1%	0.29
Digitoxin	5%	9.1%	0.46

*SR* single-chamber pacemaker, *DR* double-chamber pacemaker, medication: *ACEI* angiotensin-converting enzyme inhibitor, *ARB* angiotensin receptor blocker, *MRA* mineralocorticoid receptor antagonist, *BB* beta-blocker, *CCB* calcium canal blocker

Post-PPM implantation patients’ management was similar in the two groups, including pharmacological management at discharge and follow-up (Table 2).

From a clinical standpoint, all patients were alive at follow-up and none had reported major adverse cerebral and cerebrovascular events. Although New York Heart Association (NYHA) class distribution was similar, there was a trend for higher NYHA class rate (III–IV) in patients with PICM (NYHA III–IV 40.5% vs. 20.5%; *p* = 0.1).

The multivariate logistic regression model to chase independent determinants for PICM included mainly three variables, i.e., preimplantation LVEF%, the presence of pre-procedural SSS, and the presence of atrioventricular cardiac conduction block of second degree or higher.

Only preimplantation LVEF% (OR = 0.88; CI 0.80–0.96; *p* = 0.006) and the presence of SSS (OR = 0.1; CI 0.03–0.9; *p* = 0.04) were independently related (inverse relationship) to follow-up PICM (Table 3).

**Table 3:** Multivariable analysis: dependent variable: pacemaker-induced cardiomyopathy (PICM)

	*p* value	OR	95% CI
SSS	0.04	0.1	0.03–0.9
AV block 2	0.7	1.3	0.2–5.9
LVEF%	0.006	0.88	0.8–0.9

*SSS* sick sinus syndrome, *AV block 2* second-degree atrioventricular conduction block, *LVEF%* left ventricular ejection fraction, *OR* odds ratio, *CI* confidence interval

## Discussion

Our study confirms that (1) in patients with no initial evidence of structural heart disease and with preserved LVEF% there is a non-negligible 6% rate of new-onset PICM at midterm follow-up after implantation of a RVA PPM; (2) even though our entire patients’ cohort included only patients with preserved LVEF% at the time of RVA PPM implant, baseline LVEF% has an independent impact upon PICM occurrence (inverse relationship) at midterm follow-up; (3) the indication for PPM implant and the pacing time rate are both important factors determining the occurrence of PICM.

Although there is evidence that in the overall population of patients treated with PPM conventional RVA pacing may result in adverse LV remodeling and consequently in a LVEF% overall reduction at midterm and longer-term follow-up [9–16], the present literature concerning the effects of chronic RVA pacing specifically on patients with preserved LVEF% and no other possible cause of CM is limited and often contradictory [16, 17]. More importantly, it is not clear how and when the negative LV remodeling will lead to PICM and heart failure symptoms. In this context, the real and independent impact of RVA pacing upon LVEF% is difficult to detect and is often influenced by pre-procedural confounding factors. This is partly due to the fact that it is not always practical to perform an adequate patients’ selection and identify only those patients with preserved LVEF% that, after PPMI, develop fatal heart failure, in the clear absence of concomitant causes of CM, apart from PICM.

Furthermore, observation of a significant reduction in LVEF% after PPMI does not necessarily imply development of PICM and, for this reason, a very specific and imaging-based definition of PICM should be given with the goal to clarify the realistic effects of chronic RVA pacing.

The effects of sole RVA pacing have been investigated thoroughly using, as control groups, patients implanted and stimulated with bi-ventricular (RV and LV) systems. In a series of prospective randomized studies, it emerges that the detrimental effects of pacing are significantly more marked in patients undergoing sole RVA pacing and not significant alterations of the LVEF% have been reported in cardiac resynchronization therapy (CRT) patients [9]. The Pacing to Avoid Cardiac Enlargement (PACE) trial reported, in patients with preserved LVEF%, the 2-year follow-up superiority of bi-ventricular pacing to RVA pacing in the prevention of LV adverse remodeling and deterioration of systolic function [16]. The rate of PICM after RVA pacing can be higher than 15% at 2-year follow-up [16].

Similar rates have been shown at longer-term follow-up (over 12 years) in smaller patients’ cohorts treated with sole RVA PPM [12]. In preventing ventricular dysfunction in pacemaker patients without advanced heart failure multicenter international randomized trial (PREVENT-HF), chronic RV pacing had no effect on the 12-month follow-up LVEF% [17]. The trial did not demonstrate significant LV volume differences between RVA and CRT pacing for atrioventricular (AV) block [17].

In our present study, we have tried to make clear about the real impact of RVA pacing by starting from a very timely selection of the study cohort and by adopting a very strict and reproducible definition of PICM to be documented within serial echocardiographic investigations at follow-up.

Starting from our overall cohort of PPM-implanted patients during the proposed study period, less than a fourth of the patients were actually considered as adequate candidates for the study. In fact, the majority of PPMs had been implanted in patients with additional cardiac or clinical conditions that may have impacted upon the future development of some sort of CM at follow-up shadowing, in this way, the true impact of RVA pacing. This testifies the fact that, as already emphasized, most of the patients referred for PPM nowadays have, at the time of referral, already a plethora of concomitant clinical conditions that may lead to heart failure during the follow-up period, independently by the presence of a RVA PPM. To overcome this problem, our study cohort has included, after the above-mentioned selection, only those patients with no concomitant cardiac conditions. As a result, even relatively healthier patients that required a low pacing rate, such as those for example with SSS, were enrolled.

Identification of determinants for LVEF% reduction and development of PICM may serve as a tool to select patients for bi-ventricular PPM implantation. This is of particular importance in patients with initially preserved or only slightly reduced LVEF% and could guide future decisions to implant, instead of a simple RVA PPM, a CRT-P device, even in the presence of LVEF > 35% and QRS < 120 ms.

Single-center and multicenter studies have identified a series of risk factors for RVA PICM including baseline LV dysfunction, percentage of RV pacing, intrinsic QRS and paced QRS duration, patient’s age, and male gender [18].

It is logical to hypothesize that baseline LVEF% might impact the cardiac prognosis of patients receiving RVA PPM. Using meta-regression analysis of previously published prospective randomized trials, Lu et al. have observed no significant relationship between baseline LVEF% and mortality or heart failure hospitalization of patients implanted with either bi-ventricular or RVA PPM [9]. The authors have concluded that, regardless of the baseline LVEF%, bi-ventricular PPM is superior to RVA PPM [9]. In contrast, studies such as the DAVID trial have clearly shown that the deleterious effects of RVA PPM are particularly evident in patients with severely reduced LVEF% (< 40%) [5]. In the more recent MOST 6-year trial, Sweeney et al. have shown that reduced LVEF% predicts sudden cardiac death and heart failure occurrence in patients with sinus node disease implanted with RVA PPM [19].

We confirm that in our overall cohort there is a significant reduction in LVEF% after RVA PPM. Actually, the mean LVEF% decreased from 66.7 to 63.2%, which still is within a normal range. The LVEF% reduction observed at our midterm follow-up is in fact less than that observed in the PACE trial [16] (2-year results) where the starting LVEF% of patients undergoing sole RVA PPM was 61.5% and was reduced to 53.0% after 2 years [16]. We have confirmed that even in patients with preserved cardiac function, baseline LVEF% will have an independent impact in the occurrence of PICM. More specifically, although patients developing PICM had on average only a 10% lower LVEF%, they experienced a 10 times higher reduction in LVEF% at 2-year follow-up after RVA PPM implantation.

Percentage of RV pacing and mode of pacing may also impact upon PICM development. In Khurshid et al. experience, PICM developed in approximately 20% of patients and was observed with an RV pacing time as little as 20% [20]. Similar findings have been confirmed more recently by other authors [21].

Although in our experience patients experiencing PMIC had a trend for a higher RV pacing time, this variable was not introduced in the multivariable model. Instead, the presence of SSS as a primary indication for PPM was inversely and independently related to PICM occurrence. This finding confirms indirectly the relationship between RV pacing time and PICM occurrence in our highly selected cohort of patients with initially preserved LVEF%. Moreover, although paced QRS duration (150–180 ms.) has been confirmed as an important predictor of PICM [18, 20, 21], we have not systematically collected QRS duration in our study and we cannot confirm this finding. Finally, it should be remarked that the development of PICM, according to the pre-specified definition used in this manuscript as in others (LVEF ≤ 45%), does not necessarily imply occurrence of heart failure symptoms. As shown by Khurshid et al., only half of the patients with PICM echocardiographic diagnosis will present clinical evidence of overt heart failure symptoms [20]. In this context, we confirm that, in spite of the fact that there was a trend for lower NYHA class at follow-up of patients with PICM, in our analysis none of these patients experienced readmission for heart failure and/or major cardiac morbidity.

## Limitations

The present study has a series of not surmountable limitations including the relatively small sample size, the relatively contained rate of PICM (that has limited the power of our multivariate analysis), and the lack of information concerning the QRS duration pre- and post-PPM.

## Data Availability

The corresponding author will make the dataset available (SPSS spreadsheet) upon request.

## Abbreviations

LBBB: left bundle branch block
RV: right ventricle
RVA: right ventricular apex
LVEF: left ventricular ejection fraction
LV: left ventricle
PPM: permanent pacemaker
PICM: pacemaker-induced cardiomyopathy
SSS: sick sinus syndrome
CRT: cardiac resynchronization therapy
AV: atrioventricular
